# Meta-analysis in periprosthetic joint infection: a global bibliometric analysis

**DOI:** 10.1186/s13018-020-01757-9

**Published:** 2020-07-10

**Authors:** Cheng Li, Christina Ojeda-Thies, Chi Xu, Andrej Trampuz

**Affiliations:** 1 Charité–Universitätsmedizin Berlin, corporate member of Freie Universität Berlin, Humboldt-Universität zu Berlin, and Berlin Institute of Health, Center for Musculoskeletal Surgery (CMSC), Charitéplatz 1, D-10117 Berlin, Germany; 2 Hospital Universitario 12 de Octubre, Madrid, Spain; 3 Department of Orthopaedic Surgery, General Hospital of People’s Liberation Army, Beijing, People’s Republic of China

**Keywords:** Bibliometrics, Arthroplasty, Surgical site infections, Periprosthetic joint infection, Meta-analysis, Research

## Abstract

**Background:**

Periprosthetic joint infection (PJI) is the most serious complication of joint replacement surgery. Further comorbidities include bedsore, deep vein thrombosis, reinfection, or even death. An increasing number of researchers are focusing on this challenging complication. The aim of the present study was to estimate global PJI research based on bibliometrics from meta-analysis studies.

**Methods:**

A database search was performed in PubMed, Scopus, and Web of Science. Relevant studies were assessed using the bibliometric analysis.

**Results:**

A total of 117 articles were included. The most relevant literature on PJI was found on Scopus. China made the highest contributions to global research, followed by the USA and the UK. The institution with the most contributions was the University of Bristol. The journal with the highest number of publications was The Journal of Arthroplasty, whereas the Journal of Clinical Medicine had the shortest acceptance time. Furthermore, the top three frequently used databases were Embase, MEDLINE, and Cochrane. The most frequent number of authors in meta-analysis studies was four. Most studies focused on the periprosthetic hip and knee. The alpha-defensin diagnostic test, preventive measures on antibiotics use, and risk factors of intra-articular steroid injections were the most popular topic in recent years.

**Conclusion:**

Based on the results of the present study, we found that there was no single database that covered all relevant articles; the optimal method for bibliometric analysis is a combination of databases. The most popular research topics on PJI focused on alpha-defensin, antibiotic use, risk factors of intra-articular steroid injections, and the location of prosthetic hip and knee infection.

## Introduction

Periprosthetic joint infection (PJI) is a serious and challenging complication after joint replacement. Due to the lack of consensus on the management of PJI, physicians often face uncertainty. However, errors in diagnosis and treatment result in increased healthcare costs, reinfection, or mortality [1]. Publications play an essential role in guiding and improving disciplinary development. Bibliometric analysis is a widely used tool that uses mathematical and statistical methods to assess research trends and growth. Another commonly used tool is meta-analysis, a statistical method of collecting and analyzing results from multiple studies to find or prove the viewpoint or relationship between variables. These two methods have been applied extensively in orthopedic research [2–6]; however, there were few publications on the use of meta-analysis in bibliometric studies [7, 8]. To date, no such studies have been performed on orthopedic research.

The choice of database and the search strategy used are a crucial step in bibliometric studies and meta-analysis. Due to differences in exporting information between different databases, most bibliometric studies use a single database for statistics and data analysis [9, 10]. Such differences regarding PJI research remained unknown. Accordingly, the present study performed a bibliometric analysis to determine the following: (1) the most suitable database (PubMed, Scopus, Web of Science) for bibliometric analysis [11]; (2) global research characteristics of PJI through the analysis of meta-analysis publications; (3) countries with the most research on the meta-analysis of PJI; (4) the diagnostic method with the highest sensitivity preoperatively, intraoperatively, and before reimplantation based on meta-analysis results; (5) the effective prevention measurement or risk factor on the meta-analysis of PJI; and (6) conclusions supported by the current meta-analysis.

## Materials and methods

### Data sources and searches

We systematically searched PubMed, Scopus, and Web of Science from inception to December 2019. The search algorithm used was the following medical subject headings (MeSH) or keywords: “arthroplasty”, “joint prosthesis”, “joint replacement”, “periprosthetic joint”, “prosthetic joint”, “infection”, “infectious”, “infected”, “meta analysis”, and “meta-analysis”. As this study was performed using global research, there were no language restrictions.

### Data collection

Data were extracted independently by two reviewers (LC and COT). Discrepancies were adjudicated by the third author (XC). Information on all eligible publications including the title, author, year of publication, country, institution, journal, keywords, citations, state of the manuscript, language, number of studies, impact factor, software, database, search algorithm, and subject information were collected. The number of citations was based on the final result, in the case that no single database covered all citation information. Subsequently, citations were collected from Google scholar. Finally, two authors (LC and COT) manually screened and analyzed the publication information in Microsoft Excel (Microsoft, Redmond, Washington, USA, 2010) and EndNote X7 (Thomson Reuters, New York, NY, USA, 2013).

## Results

### Database results

**Fig. 1:**
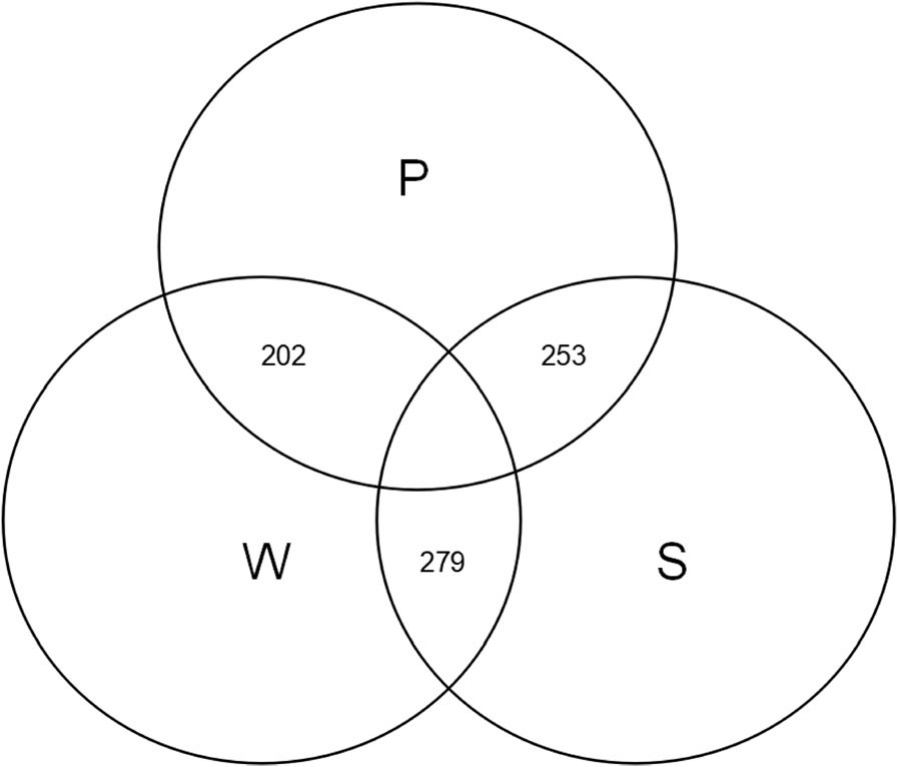
Number of shared duplicate articles between the three databases

**Fig. 2:**
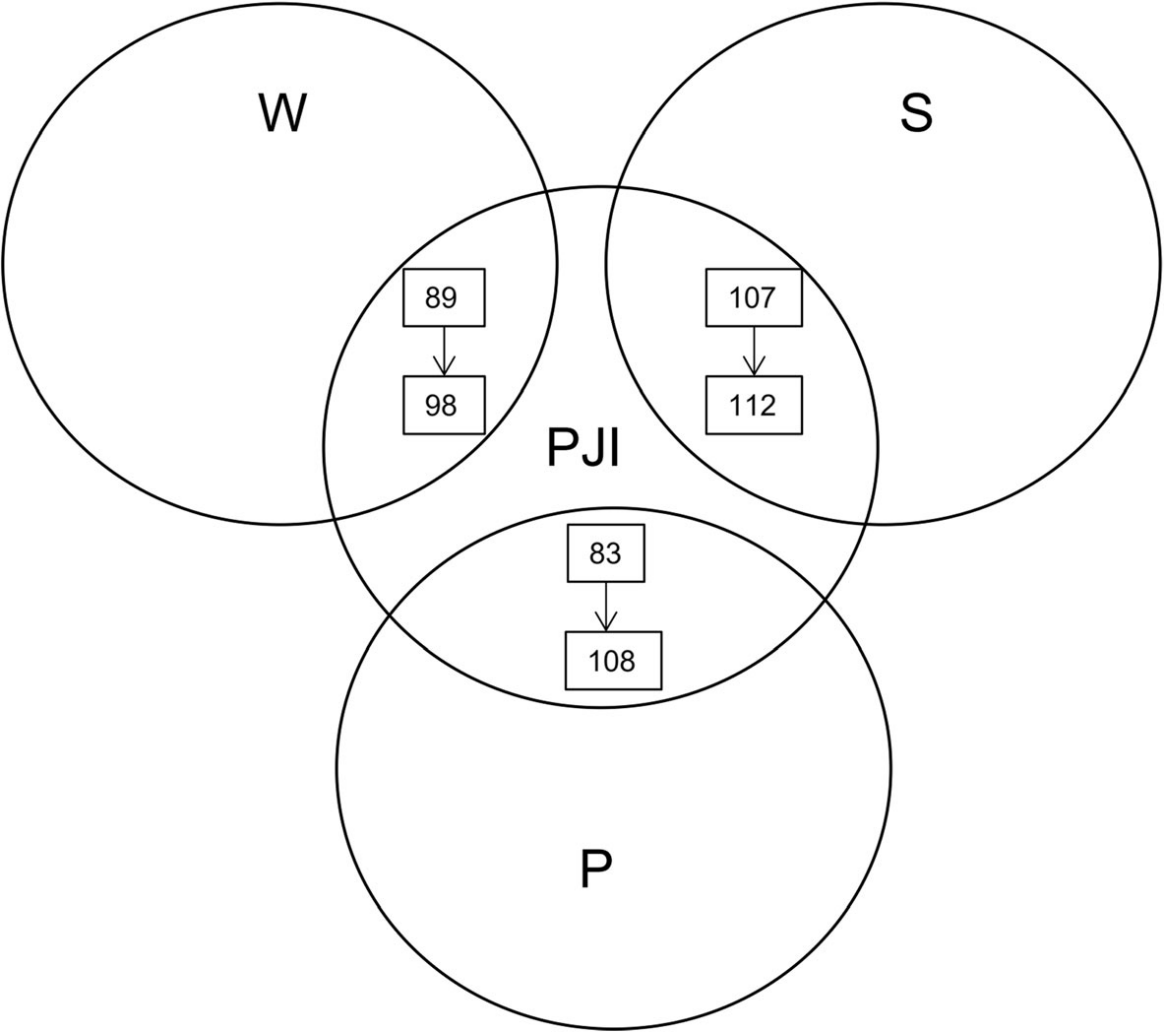
Number of shared PJI research articles of meta-analysis between the three databases (with or without search algorithms)

**Fig. 3:**
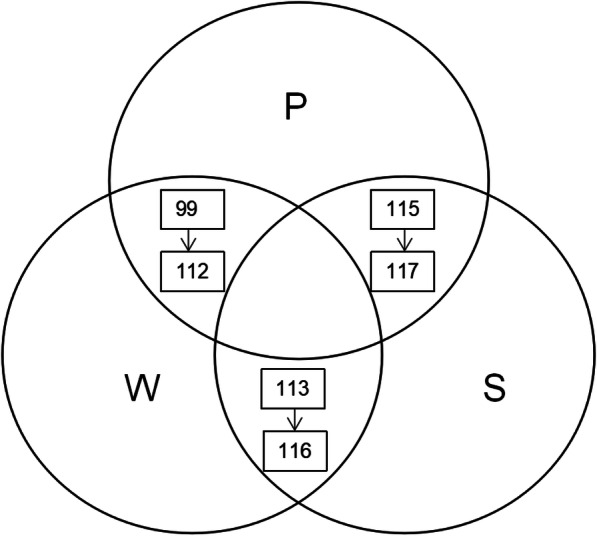
Number of shared meta-analysis of PJI research in the combined databases (with or without search algorithms)

Results from the search strategy demonstrated that the database with the most publications was Scopus (570), followed by Web of Science (341), and PubMed (243). The greatest number of identical articles was through the combined database of Web of Science and Scopus (Fig. 1). Finally, a total of 117 related articles were included. Of these, the database with most publications on the meta-analysis of PJI was Scopus, followed by Web of Science and PubMed. Web of Science and PubMed had most missed articles compared with other databases (Figs. 2 and 3).

### Characteristics of meta-analysis of PJI research

#### General data

**Fig. 4:**
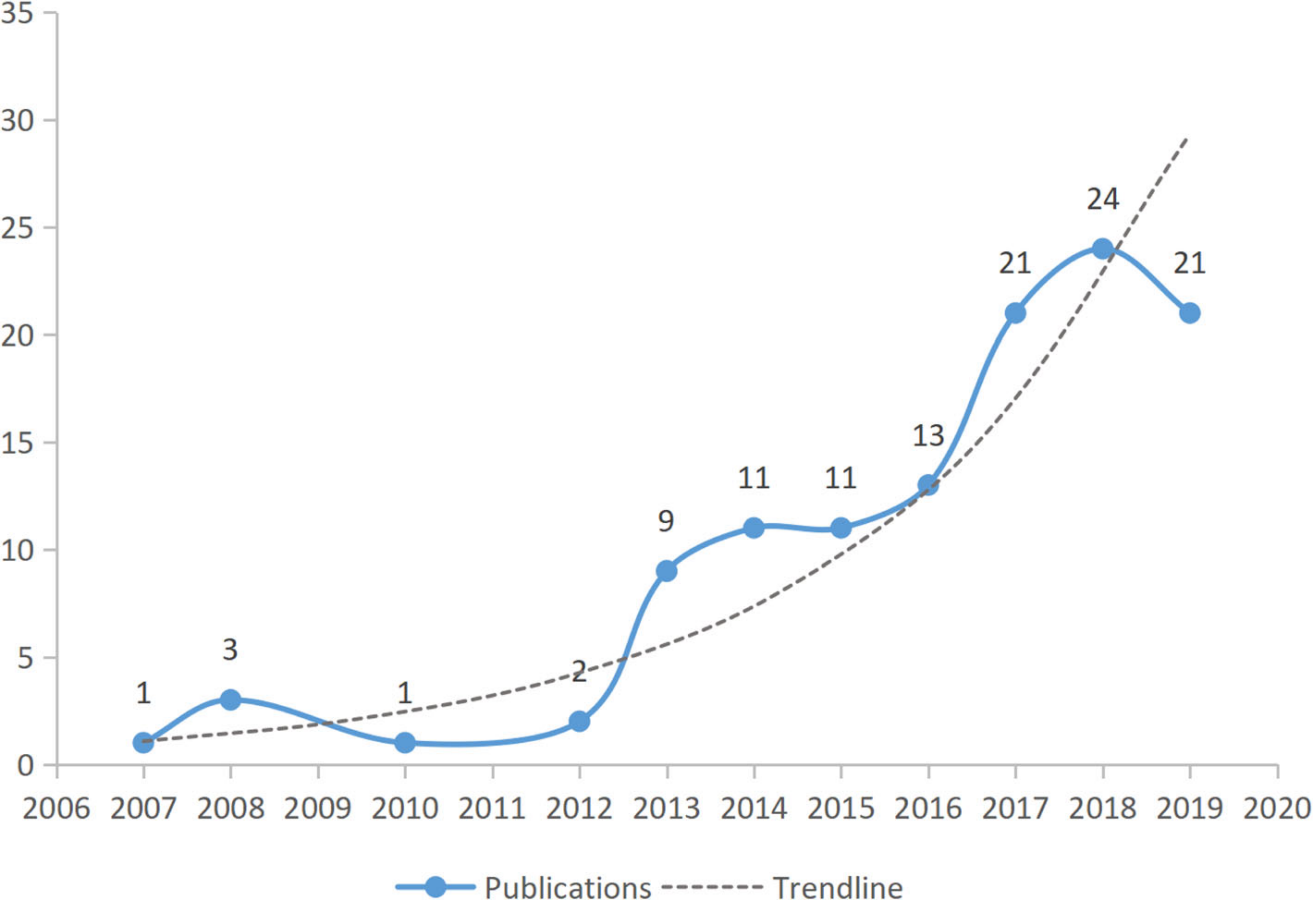
Total annual number of publications and trendline in the meta-analysis of PJI

Among the 117 meta-analysis articles, the earliest publications were from 2007. The greatest number of articles were published in 2018 (24), followed by 2017 and 2019 (21 each). The trend line indicates an annual increase in the number of articles (Fig. 4). One hundred and fourteen articles were in English, and three other articles were each published in Chinese, German, and Persian. In all meta-analyses, the number of studies included ranged from 4 to 203, with the highest number 12 (*n* = 11 publications), followed by eight (9) as well as six and eleven studies (8 each).

#### Countries

**Fig. 5:**
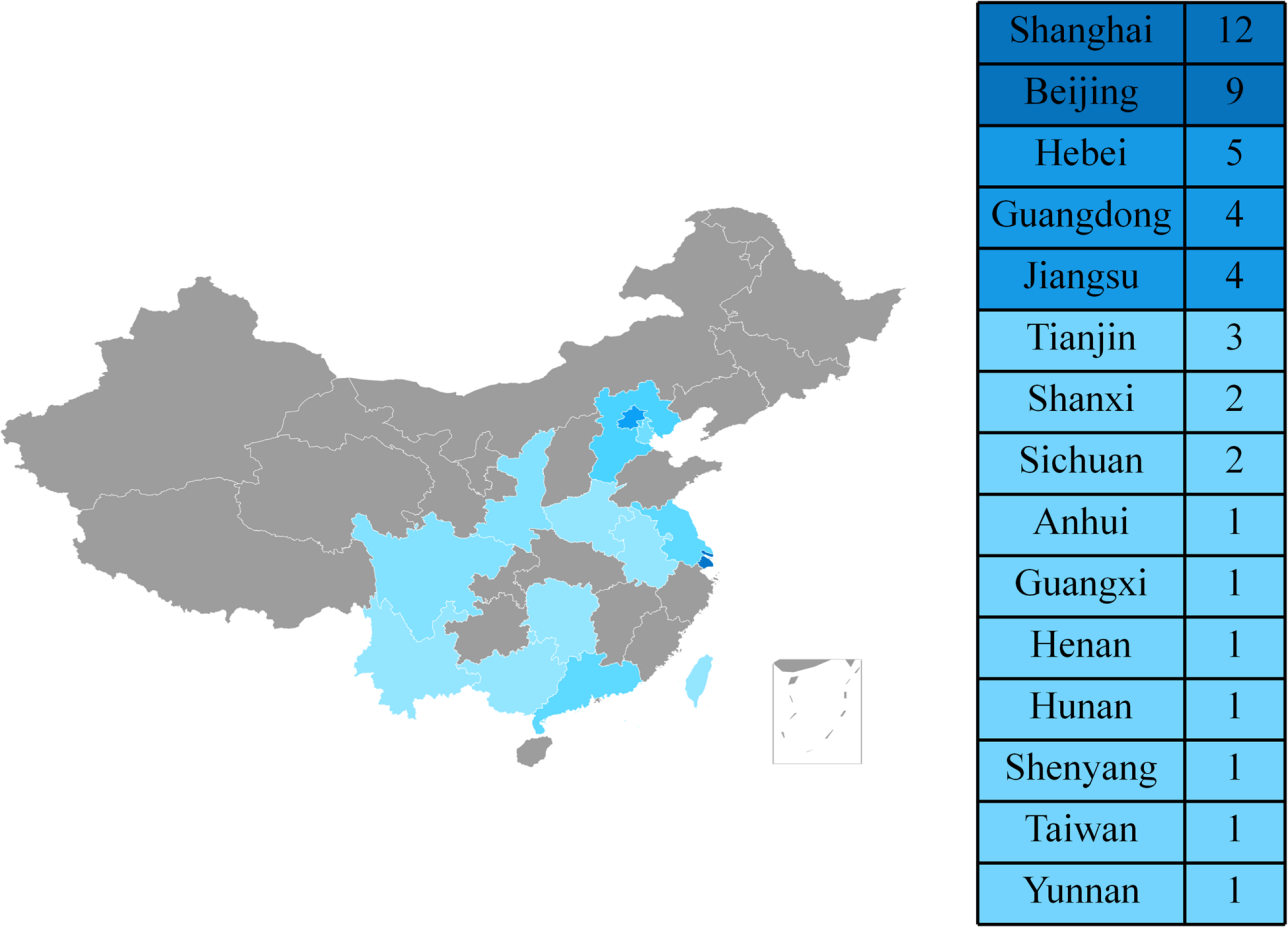
Map showing the distribution of meta-analysis studies on PJI from China

Nineteen countries published meta-analyses on PJI. Of these, China was the most productive country, with all publications stemming from 15 cities/provinces. The highest number of articles originated from Shanghai, followed by Beijing (Fig. 5). The country with the second highest number of publications on PJI was the US, followed by the UK (Table 1).

**Table 1:** Global distribution of meta-analysis studies on PJI

Country	Number of articles
China	48
USA	20
UK	18
Germany	4
South Korea	4
Italy	3
Netherlands	3
Canada	3
Australia	2
Colombia	2
Greece	2
Brazil	1
Denmark	1
Iran	1
Portugal	1
South Africa	1
Spain	1
Sweden	1
Switzerland	1

#### Institutions

A total of 76 institutions made contributions to this field. The institution with the greatest number of publications was the University of Bristol with 11 papers, followed by Shanghai Sixth People’s Hospital (8). The Rothman Institute and General Hospital of the Chinese People’s Liberation Army were third, with each publishing five research articles. Fourteen institutions published more than one paper, with 50% originating from China (Table 2).

**Table 2:** Top 14 institutions and countries of meta-analysis studies on PJI

Name of institution	Publication	Country
University of Bristol	11	UK
Shanghai Sixth People’s Hospital	8	China
Rothman Institute	5	USA
General Hospital of Chinese People’s Liberation Army	5	China
The Third Hospital of Hebei Medical University	4	China
Tianjin Hospital	3	China
Mayo Clinic Hospital	3	USA
Charité - Universitätsmedizin Berlin	3	Germany
West China Hospital	2	China
Medical Centre Alkmaar	2	Netherlands
McMaster University	2	Canada
Federico II University	2	Italy
Beijing Jishuitan Hospital	2	China
Beijing Friendship Hospital of Capital Medical University	2	China

#### Authors

The number of authors of a single article ranged from 2 to 37. The largest number of collaborating authors was four (27), followed by six (25) and 5 authors (20; Table 3). The author with most first authorships was Setor K. Kunutsor (10), followed by Xinhua Qu (3). Ten first authors wrote more than one meta-analysis, with 50% published by research institutes in China (Table 4).

**Table 3:** Number of collaborating authors in meta-analysis studies on PJI

Number of authors	Total number
4	27
6	25
5	20
3	15
7	10
8	6
10	3
2	2
9	2
13	2
27	2
11	1
18	1
37	1

**Table 4:** List of top 10 first authors with number of publications and institution of meta-analysis studies on PJI.

First author	Publications	Institution
Setor K. Kunutsor	10	University of Bristol
Qu Xinhua	3	Shanghai Ninth People’s Hospital
Giovanni Balato	2	Federico II University
Yong Seuk Lee	2	Rothman Institute
Li Cheng	2	Charité Universitätsmedizin Berlin
Steven J. Verberne	2	Medical Centre Alkmaar
Wang Chi	2	General Hospital of Chinese People’s Liberation Army
Xu Chi	2	General Hospital of Chinese People’s Liberation Army
Dan Xing	2	Tianjin Hospital
Xie Kai	2	Shanghai Ninth People’s Hospital

#### Journals

Meta-analysis studies were published in 54 different journals. The journal with most publications was the Journal of Arthroplasty, with 15 publications. The Journal of Bone and Joint Surgery ranked second with eight publications, whereas PLoS ONE was third with seven. Nineteen journals had more than one publication (Table 5). In 2019, an impact factor was available for 42 journals. The list of top 10 journals with the highest impact factors is shown in Table 6.

**Table 5:** Top 19 journals with number of publications and their corresponding impact factor of meta-analysis studies on PJI

Journal	Number of publications	Impact factor
Journal of Arthroplasty	15	3.524
Journal of Bone and Joint Surgery-American Volume	8	4.716
PLoS ONE	7	2.776
Surgical Infections	6	1.921
International Orthopaedics	5	2.384
Bone and Joint Journal	5	4.301
Archives of Orthopaedic and Trauma Surgery	3	1.973
BMC Musculoskeletal Disorders	3	2.002
Clinical Orthopaedics and Related Research	3	4.154
International Journal of Clinical and Experimental Medicine	3	0.181
Journal of Clinical Microbiology	3	4.959
Journal of Hospital Infection	3	3.704
Journal of Orthopaedic Surgery and Research	3	1.907
Knee Surgery Sports Traumatology Arthroscopy	3	3.149
Medicine	3	1.87
Orthopedics	3	1.608

**Table 6:** List of top 10 highest impact factor journals with number of PJI publications in meta-analysis

Journal	Number of publications	Impact factor
Radiology	1	7.608
European Journal of Nuclear Medicine and Molecular Imaging	1	7.182
European Journal of Epidemiology	1	6.529
Journal of Clinical Medicine	2	5.688
Journal of Antimicrobial Chemotherapy	1	5.113
Regional Anesthesia and Pain Medicine	1	5.113
Journal of Infection	1	5.099
Journal of Clinical Microbiology	3	4.959
Journal of Bone and Joint Surgery-American Volume	8	4.716
Antimicrobial Agents and Chemotherapy	1	4.715

From all publications, the date of receipt was available for 89 papers, whereas the date of acceptance for 85, and the date of publication for 72. From the date of receipt to acceptance, information was available for 65 articles, with the average number of days until acceptance 95.69. Among these 65 articles, 11 journals had more than two publications, whereas four journals had an average acceptance time of fewer than 100 days. These are the Journal of Orthopaedic Surgery and Research (68 days), followed by the Journal of Hospital Infection (82 days), Journal of Clinical Microbiology (83 days), and Journal of Arthroplasty (86 days).

The average number of days from acceptance to publication was 56.52 (66 papers). From receipt to online publication, the average number of days was 157.48 (69). There were six articles accepted in less than 30 days after submission. The journal with the shortest acceptance time was the Journal of Clinical Medicine (16 days), followed by the Journal of Computational and Theoretical Nanoscience (18 days), Journal of Clinical Medicine (22 days), Journal of Arthroplasty (23 days), Journal of Orthopaedic Surgery and Research as well as Medical Science Monitor (27 days each).

#### Most cited publications

From Google Scholar, citation information was available for 103 meta-analyses. Forty-one articles were cited more than 20 times, with the highest number in 2014 (9), followed by 2013, 2016, and 2017 (7 each). The most cited article was published by AlBuhairan et al. [12] (264), followed by Parvizi et al. [13] (235; Table 7).

**Table 7:** The 50 most cited meta-analysis studies on PJI ranked by citation.

Rank	Title	Times cited
1	Antibiotic prophylaxis for wound infections in total joint arthroplasty: a systematic review	264
2	Efficacy of antibiotic-impregnated cement in total hip replacement: a meta-analysis	235
3	Incidence and risk factors for surgical site infection following total knee arthroplasty: a systematic review and meta-analysis	172
4	Utility of intraoperative frozen section histopathology in the diagnosis of periprosthetic joint infection: a systematic review and meta-analysis	145
5	FDG-PET for diagnosing prosthetic joint infection: systematic review and metaanalysis	144
6	Patient-related risk factors for periprosthetic joint infection after total joint arthroplasty: a systematic review and meta-analysis	139
7	Chronic infections in hip arthroplasties: comparing risk of reinfection following one-stage and two-stage revision: a systematic review and meta-analysis	117
8	Risk factors for periprosthetic joint infection after total joint arthroplasty: a systematic review and meta-analysis	114
9	The alpha-defensin immunoassay and leukocyte esterase colorimetric strip test for the diagnosis of periprosthetic infection a systematic review and meta-analysis	90
10	A systematic review and meta-analysis of antibiotic-impregnated bone cement use in primary total hip or knee arthroplasty	88
11	Risk factors for deep infection after total knee arthroplasty: a meta-analysis	81
12	Infection after primary total hip arthroplasty	79
13	Re-infection outcomes following one- and two-stage surgical revision of infected knee prosthesis: a systematic review and meta-analysis	78
14	Prosthesis infection: diagnosis after total joint arthroplasty with antigranulocyte scintigraphy with99mTc-labeled monoclonal antibodies - a meta-analysis	76
15	Allogeneic blood transfusion is a significant risk factor for surgical-site infection following total hip and knee arthroplasty: a meta-analysis	74
16	Inflammatory blood laboratory levels as markers of prosthetic joint infection: a systematic review and meta-analysis	74
17	Meta-analysis of sonication fluid samples from prosthetic components for diagnosis of infection after total joint arthroplasty	68
18	Re-infection outcomes following one- and two-stage surgical revision of infected hip prosthesis: a systematic review and meta-analysis	65
19	Use of static or articulating spacers for infection following total knee arthroplasty	61
20	PCR-based diagnosis of prosthetic joint infection	57
21	Preoperative aspiration culture for preoperative diagnosis of infection in total hip or knee arthroplasty	56
22	Synovial fluid biomarkers for the diagnosis of periprosthetic joint infection: a systematic review and meta-analysis	47
23	Evaluation of white cell count and differential in synovial fluid for diagnosing infections after total hip or knee arthroplasty	35
24	Prosthesis infection: diagnosis after total joint arthroplasty with three-phase bone scintigraphy	35
25	Diagnostic performance of FDG PET or PET/CT in prosthetic infection after arthroplasty: a meta-analysis	34
26	Procalcitonin and a-defensin for diagnosis of periprosthetic joint infections	34
27	The accuracy of imaging techniques in the assessment of periprosthetic hip infection: a systematic review and meta-analysis	32
28	Control strategies to prevent total hip replacement-related infections: a systematic review and mixed treatment comparison	30
29	Outcomes following debridement, antibiotics and implant retention in the management of periprosthetic infections of the hip: a review of cohort studies	30
30	Total joint arthroplasty following intra-articular steroid injection: a literature review	30
31	Do intra-articular steroid injections increase infection rates in subsequent arthroplasty? A systematic review and meta-analysis of comparative studies	29
32	Postoperative antibiotic prophylaxis in total hip and knee arthroplasty: a systematic review and meta-analysis of randomized controlled trials	28
33	What is the accuracy of nuclear imaging in the assessment of periprosthetic knee infection? A meta-analysis	28
34	Does previous intra-articular steroid injection increase the risk of joint infection following total hip arthroplasty or total knee arthroplasty? A meta-analysis	27
35	Systematic review and meta-analysis of randomized controlled trials of antibiotics and antiseptics for preventing infection in people receiving primary total hip and knee prostheses	25
36	Diagnostic accuracy of C-reactive protein for periprosthetic joint infection: a meta-analysis	24
37	Use of anti-granulocyte scintigraphy with 99mTc-labeled monoclonal antibodies for the diagnosis of periprosthetic infection in patients after total joint arthroplasty: a diagnostic meta-analysis	24
38	Serum and synovial fluid interleukin-6 for the diagnosis of periprosthetic joint infection	23
39	The application of sonication in diagnosis of periprosthetic joint infection	22
40	The impact of neuraxial versus general anesthesia on the incidence of postoperative surgical site infections following knee or hip arthroplasty a meta-analysis	22
41	Do ‘Surgical Helmet Systems’ or ‘Body Exhaust Suits’ affect contamination and deep infection rates in arthroplasty? A systematic review	21

#### Search algorithm and keywords

One hundred and two meta-analyses were retrieved from the search strategy, which were exported to Microsoft Excel. All keywords or MeSH were combined. PJI-related keywords were 196, followed by diagnosis (179), prevention (82), risk factor (74), and outcome (60). All keywords are presented in Supplementary 1. From 71 publications, 389 keywords were exported. Periprosthetic joint infection (41) was the most commonly used keyword, followed by meta-analysis (29) and total knee arthroplasty (20; Table 8).

**Table 8:** List of top 10 keywords of PJI publications in meta-analysis

Keywords	Occurrence (***n***)
Periprosthetic joint infection	41
Meta-analysis	29
Total knee arthroplasty	20
Arthroplasty	18
Infection	17
Total hip arthroplasty	13
Two stage	10
Alpha-defensin	9
Total joint arthroplasty	9
Knee	9

#### Database and software

After combining all databases from 116 articles, there were a total of 52 databases. Embase was the most described database (101), followed by MEDLINE (80), and Cochrane (74; Table 9). Three databases were most frequently searched (40), followed by four (22), and five (16). The most combined database group was Cochrane Library + Embase + MEDLINE/PubMed (10), followed by Embase + MEDLINE (6), and Cochrane Library + Embase + MEDLINE + Web of Science (5).

**Table 9:** List of top 10 databases of PJI in meta-analysis

Database	Occurrence (***n***)
Embase	101
MEDLINE	80
Cochrane	74
PubMed	57
Web of Science	36
OVID	14
Scopus	14
Science Direct	12
Google Scholar	9
CNKI	8

For the meta-analysis, 13 softwares were exported from 106 articles. The most commonly used software was STATA (43), followed by REVMAN (25), and Meta-Disc (21).

#### Subject

##### Location

Information on the site of prosthetic joint infection from the included meta-analysis were found in 112 papers. The location with the highest number was the knee (93), closely pursued by the hip (90), shoulder (23), elbow (16), and ankle (3).

##### Diagnosis of PJI

**Fig. 6:**
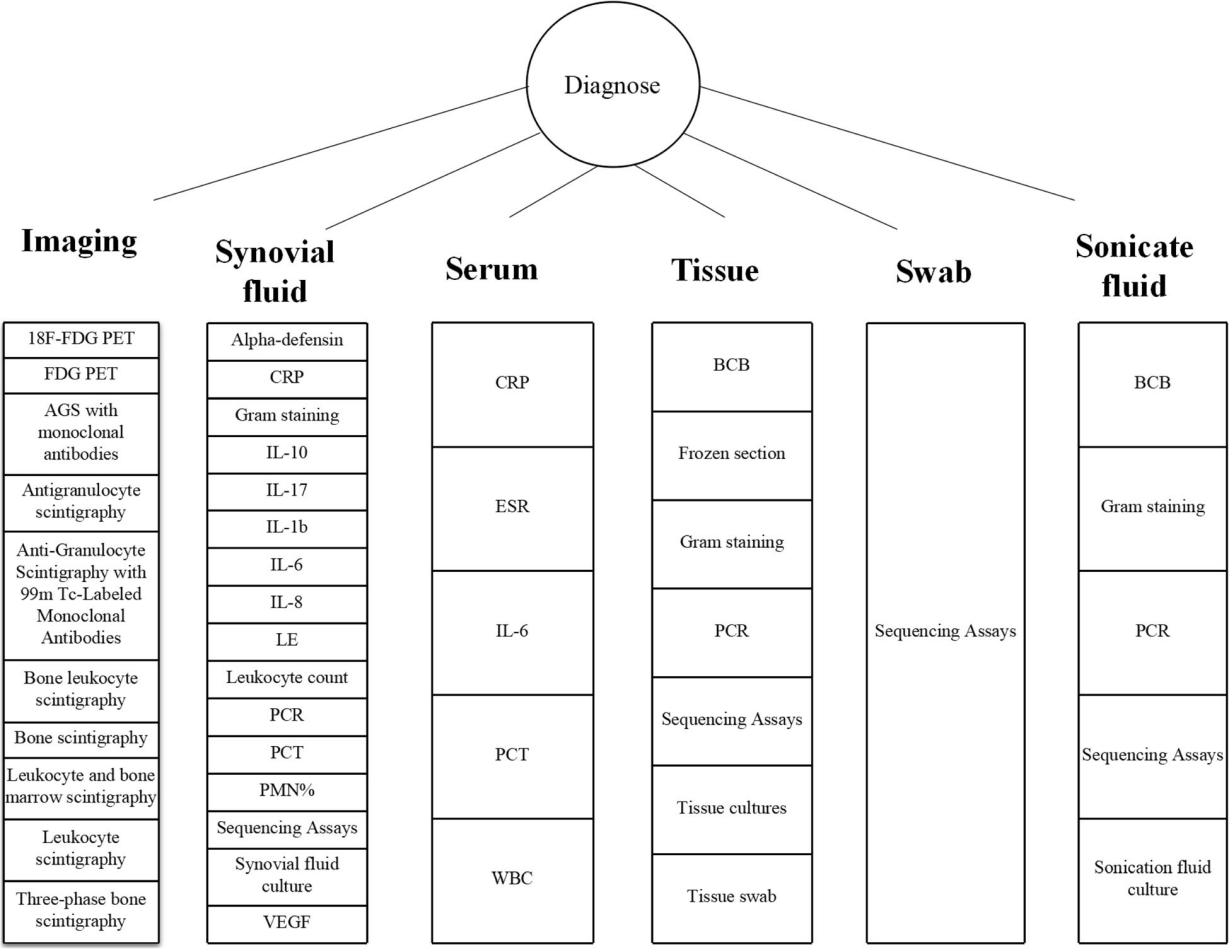
Diagnostic methods from different samples used

From 40 diagnosis-related meta-analyses, 72 tests were related to preoperative examination, followed by intraoperative methods (12), and test prior to reimplantation (14). Synovial fluid alpha-defensin had highest pooled sensitivities in the list of preoperative examinations, pursued by serum IL-6 and bone scintigraphy. From all intraoperative examinations, tissue polymerase chain reaction (PCR) was the most sensitive method, followed by sonicate fluid into blood culture bottles (BCB) and PCR. Tissue culture was the most sensitive method before reimplantation, followed by the percentage of polymorphonucleocytes in synovial fluid (PMN%), and synovial fluid culture (Table 10). The most frequent diagnostic method used was synovial fluid (16), followed by imaging (10), and periprosthetic tissue (7; Fig. 6).

**Table 10:** Diagnostic methods used for PJI detection ranked by the sensitivity (preoperative examination, intraoperative methods, and test before reimplantation)

	Reference	Year	No. of studies	Sen (95% CI)	Spe (95% CI)
**Preoperative examination**					
Synovial fluid alpha-defensin immunoassay	[14]	2016	6	1.00 (0.82–1.00)	0.96 (0.89–0.99)
Synovial fluid ELISA	[15]	2018	4	0.98 (0.94–1.00)	0.97 (0.95–0.99)
Synovial fluid alpha-defensin immunoassay	[16]	2019	7	0.98 (0.94–0.99)	0.96 (0.94–0.98)
Synovial fluid a-defensin	[17]	2017	7	0.97 (0.93–0.99)	0.96 (0.94–0.98)
Serum IL-6	[18]	2010	3	0.97 (0.93–0.99)	0.91 (0.87–0.94)
Synovial fluid ELISA	[19]	2018	4	0.97 (0.91–0.99)	0.97 (0.94–0.98)
Synovial fluid alpha-defensin immunoassay	[20]	2018	4	0.96 (0.90–0.98)	0.96 (0.93–0.97)
Synovial fluid alpha-defensin	[21]	2017	11	0.96 (0.87–0.99)	0.95 (0.91–0.97)
Synovial fluid alpha-defensin	[22]	2016	6	0.96 (0.85–0.99)	0.95 (0.89–0.98)
Synovial fluid alpha-defensin immunoassay	[23]	2018	7	0.95(0.87–0.98)	0.97 (0.94–0.98)
Synovial fluid ELISA	[24]	2018	4	0.95 (0.91–0.98)	0.97 (0.95–0.98)
Bone scintigraphy	[25]	2017	6	0.93 (0.85–0.98)	0.56 (0.47–0.64)
Synovial fluid CRP	[26]	2016	6	0.92 (0.86–0.96)	0.90 (0.87–0.93)
Synovial fluid ELISA	[27]	2019	4	0.92 (0.86–0.96)	0.99 (0.98–1.00)
Synovial fluid LE	[28]	2015	4	0.92(0.86–0.96)	0.95 (0.93–0.97)
Synovial fluid PMN%	[29]	2018	10	0.91 (0.87–0.93)	0.86 (0.81–0.90)
Synovial fluid IL-6	[30]	2017	8	0.91 (0.82–0.96)	0.90 (0.84–0.95)
Synovial fluid WCC/PMN%	[31]	2014	9	0.91 (0.82–0.95)	0.89 (0.81–0.94)
Synovial fluid WBC	[29]	2018	10	0.90 (0.87–0.92)	0.90 (0.81–0.95)
Synovial fluid PMN%	[31]	2014	14	0.90 (0.84– 0.93)	0.88 (0.83–0.92)
AGS	[25]	2017	5	0.90 (0.78–0.96)	0.95 (0.88–0.98)
Synovial fluid LE	[32]	2018	8	0.90 (0.76–0.96)	0.97 (0.95–0.98)
Synovial fluid leukocyte count	[17]	2017	12	0.89 (0.86–0.91)	0.86 (0.80–0.90)
Synovial fluid PMN%	[17]	2017	10	0.89 (0.82–0.93)	0.86 (0.77–0.92)
Serum CRP	[18]	2010	23	0.88 (0.86–0.90)	0.74 (0.71–0.76)
Synovial fluid WCC	[31]	2014	15	0.88 (0.81–0.93)	0.93 (0.88–0.96)
Leukocyte scintigraphy	[25]	2017	6	0.88 (0.81–0.93)	0.77 (0.69–0.85)
Leukocyte scintigraphy	[33]	2016	6	0.88 (0.81– 0.94)	0.92 (0.88–0.96)
18F-FDG PET or PET/CT	[34]	2017	16	0.87(0.83–0.90)	0.87 (0.85–0.89)
Serum CRP	[35]	2017	11	0.87 (0.84–0.90)	0.79 (0.77–0.80)
Bone and leukocyte scintigraphy	[25]	2017	4	0.87 (0.71–0.96)	0.82 (0.72–0.90)
Synovial fluid IL-8	[17]	2017	3	0.87 (0.67–0.96)	0.94 (0.88–0.97)
Serum ESR	[35]	2017	12	0.86 (0.83–0.89)	0.72 (0.70– 0.74)
FDG PET or PET/CT	[36]	2013	14	0.86 (0.82–0.90)	0.86(0.83–0.89)
Synovial fluid CRP	[19]	2018	9	0.86 (0.81–0.91)	0.90 (0.86–0.93)
FDG PET	[33]	2016	12	0.86 (0.80–0.90)	0.93 (0.90–0.95)
Synovial fluid lateral flow test	[27]	2019	12	0.85 (0.80–0.89)	0.96 (0.94–0.97)
Synovial fluid CRP	[17]	2017	10	0.85 (0.78–0.90)	0.88 (0.78–0.94)
Synovial fluid lateral flow test	[24]	2018	6	0.85 (0.74–0.92)	0.90 (0.91–0.98)
Synovial fluid/serum CRP	[28]	2015	15	0.845 (0.82–0.87)	0.795 (0.78–0.81)
Synovial fluid PCR	[37]	2013	6	0.84 (0.75–0.93)	0.89 (0.81–0.97)
Synovial fluid lateral flow test	[16]	2019	6	0.84 (0.74–0.91)	0.94 (0.89–0.97)
AGS	[33]	2016	5	0.84 (0.70–0.93)	0.75 (0.66–0.82)
AGS with monoclonal antibodies	[38]	2007	13	0.83(0.75–0.89)	0.80 (0.75–0.84)
Anti-granulocyte scintigraphy with 99 m Tc-labeled monoclonal antibodies	[39]	2013	19	0.83 (0.79–0.87)	0.79 (0.75–0.83)
Synovial fluid/serum IL-6	[40]	2018	18	0.83 (0.74–0.89)	0.91 (0.84–0.95)
Three-phase bone scintigraphy	[41]	2014	20	0.83 (0.72–0.90)	0.73 (0.65–0.80)
Synovial fluid/serum IL-6	[28]	2015	11	0.824 (0.78–0.87)	0.85 (0.82–0.88)
FDG-PET	[42]	2008	11	0.82(0.68–0.91)	0.87 (0.80–0.91)
Serum CRP	[43]	2014	25	0.82 (0.80–0.84)	0.77 (0.76–0.78)
Synovial fluid IL-6	[17]	2017	5	0.81 (0.70–0.89)	0.94 (0.88–0.97)
Synovial fluid LE	[14]	2016	5	0.81 (0.49–0.95)	0.97(0.82–0.99)
Bone scintigraphy	[33]	2016	8	0.80 (0.72–0.86)	0.69 (0.64–0.73)
Leukocyte and bone marrow scintigraphy	[25]	2017	7	0.80 (0.66–0.91)	0.93 (0.86–0.97)
Synovial fluid Synovasure	[19]	2018	6	0.80 (0.65–0.89)	0.89 (0.76–0.96)
Synovial fluid LE	[27]	2019	12	0.79 (0.75–0.82)	0.96 (0.95–0.97)
Synovial fluid LE	[19]	2018	12	0.79 (0.67–0.87)	0.92 (0.87–0.92)
Synovial fluid lateral flow test	[23]	2018	3	0.77 (0.64–0.87)	0.91 (0.83–0.96)
Synovial fluid LE	[17]	2017	5	0.77 (0.63–0.87)	0.95 (0.86–0.98)
Synovial fluid IL-6	[19]	2018	11	0.76 (0.65–0.84)	0.91 (0.88–0.94)
Serum ESR	[18]	2010	25	0.75 (0.72–0.77)	0.70 (0.68–0.72)
Synovial fluid culture	[44]	2013	34	0.72 (0.65–0.78)	0.95 (0.93–0.97)
Serum IL-6	[30]	2017	11	0.72 (0.63–0.80)	0.89 (0.77–0.95)
Synovial fluid lateral flow test	[20]	2018	3	0.71 (0.55–0.83)	0.90 (0.81–0.95)
FDG-PET	[25]	2017	5	0.70 (0.56–0.81)	0.84 (0.76–0.90)
Leukocyte and bone marrow scintigraphy	[33]	2016	3	0.69 (0.58–0.79)	0.96 (0.93–0.98)
Synovial fluid culture	[17]	2017	5	0.62 (0.50–0.74)	0.94 (0.91–0.96)
Serum PCT	[40]	2018	6	0.58 (0.31–0.81)	0.95 (0.63–1.00)
Serum PCT	[22]	2016	6	0.53 (0.24–0.80)	0.92 (0.45–0.99)
Serum WBC	[18]	2010	15	0.45 (0.41–0.49)	0.87 (0.85–0.89)
Synovial fluid PCT	[28]	2015	3	0.35(0.28–0.43)	0.994 (0.97–1.00)
Synovial fluid GS	[45]	2015	4	0.30 (0.17–0.48)	1.00 (0.88–1.00)
**Intraoperative examination**					
Tissue PCR	[37]	2013	5	0.95 (0.91–0.99)	0.81 (0.66–0.90)
Sonicate fluid BCB	[46]	2018	4	0.85 (0.77–0.91)	0.86 (0.81–0.91)
Sonicate fluid PCR	[37]	2013	4	0.81 (0.71–0.91)	0.96 (0.92–1.00)
Sonicate fluid	[47]	2014	12	0.80 (0.74–0.84)	0.95 (0.90–0.98)
Sonicate fluid	[48]	2017	16	0.79 (0.76–0.81)	0.95 (0.94–0.96)
Synovial fluid WCC/PMN%	[31]	2014	4	0.77 (0.51–0.91)	0.97 (0.93–0.99)
Sonicate fluid PCR	[49]	2018	9	0.75 (0.71–0.79)	0.96 (0.94–0.97)
Tissue-frozen section [five leukocytes per high power field (400×)]	[50]	2013	10	0.73 (0.65–0.80)	0.90 (0.88–0.93)
Tissue BCB	[51]	2019	4	0.70 (0.66–0.75)	0.97 (0.95–0.98)
Tissue-frozen section [ten leukocytes per high power field (400×)]	[50]	2013	5	0.64 (0.54–0.74)	0.95 (0.93–0.97)
Tissue GS	[45]	2015	5	0.16 (0.08–0.29)	0.99 (0.98–1.00)
Tissue swab GS	[45]	2015	3	0.14 (0.07–0.24)	1.00 (0.97–1.00)
**Before reimplantation**					
Tissue culture	[52]	2018	2	0.82 (0.72–0.90)	0.91 (0.89–0.95)
Synovial fluid PMN%	[52]	2018	2	0.77 (0.46–0.95)	0.74 (0.67–0.81)
Synovial fluid PMN%	[53]	2018	4	0.70 (0.58–0.81)	0.71 (0.66–0.77)
Synovial fluid culture	[52]	2018	2	0.64 (0.52–0.74)	0.96 (0.93–0.98)
Serum ESR	[53]	2018	5	0.57 (0.45–0.68)	0.50 (0.45–0.56)
Serum ESR	[52]	2018	3	0.56 (0.40–0.72)	0.60 (0.53–0.66)
Serum CRP	[52]	2018	3	0.53 (0.39–0.67)	0.72 (0.66–0.78)
Spacer sonicate fluid culture	[53]	2018	4	0.53 (0.38–0.68)	0.84 (0.76–0.90)
Synovial fluid WBC	[53]	2018	5	0.52 (0.41–0.63)	0.66 (0.61–0.71)
Serum CRP	[53]	2018	8	0.45 (0.36–0.55)	0.73 (0.69–0.77)
Synovial fluid WBC	[52]	2018	2	0.37 (0.19–0.58)	0.49 (0.41–0.57)
Tissue culture	[53]	2018	9	0.30 (0.23–0.38)	0.90 (0.87–0.92)
Frozen section	[53]	2018	4	0.29 (0.17–0.44)	0.93 (0.89–0.96)
Synovial fluid culture	[53]	2018	5	0.18 (0.11–0.28)	0.97 (0.94–0.99)

*AGS* antigranulocyte scintigraphy, *BCB* blood culture bottles, *CI* confidence interval, *CRP* C-reactive protein, *CT* computed tomography, *ELISA* enzyme-linked immunosorbent assays, *ESR* erythrocyte sedimentation, *GS* Gram staining, *LE* leukocyte esterase, *IL* interleukin, *PCR* polymerase chain reaction, *PCT* procalcitonin, *PET* positron emission tomography, *PMN%* polymorphonucleocyte percentage, *Sen* sensitivity, *Spe* specificity, *WBC* white blood cell, *WCC* white cell count

##### Risk factor and prevention

Twenty-three articles described 64 possible risk factors. The location of the risk factor was outlined in 20 studies, with the majority in the hip and knee (Table 11). Nine preventive measures were described in 17 articles, with all focusing on the hip and knee (Table 12).

**Table 11:** Risk factors of PJI based on meta-analysis studies

Title	Location	Y/N	Topic
The incidence of and risk factors for deep infection after primary shoulder arthroplasty: an updated systematic review and meta-analysis	Shoulder	Y	Male gender, avascular necrosis, rotator cuff arthropathy, proximal humerus fracture, nonunion of humerus fracture
Allogeneic blood transfusion is a significant risk factor for surgical-site infection following total hip and knee arthroplasty: a meta-analysis	Hip, knee	Y	Allogeneic blood transfusion
Association of malnutrition with periprosthetic joint and surgical site infections after total joint arthroplasty: a systematic review and meta-analysis	Hip, knee and other undefined location	Y	Malnutrition
Current evidence does not support systematic antibiotherapy prior to joint arthroplasty in patients with asymptomatic bacteriuria-a meta analysis	Hip, knee	Y	Asymptomatic bacteriuria
Do intra-articular steroid injections increase infection rates in subsequent arthroplasty? A systematic review and meta-analysis of comparative studies	Hip, knee	N	Intra-articular steroid injections
Does previous intra-articular steroid injection increase the risk of joint infection following total hip arthroplasty or total knee arthroplasty? A meta-analysis	Hip, knee	N	Intra-articular steroid injections
Dose intraarticular steroid injection increase the rate of infection in subsequent arthroplasty: grading the evidence through a meta-analysis	Hip, knee	Y	Intra-articular steroid injections
Genetic susceptibility to prosthetic joint infection following total joint arthroplasty: a systematic review	NA	Y	C allele and genotype C/C for MBL-550SNP, genotype A/A for MBL-54SNP,G allele for MBL-221SNP
Genetic susceptibility to prosthetic joint infection following total joint arthroplasty: a systematic review	NA	N	G allele and genotype G/G for MBL-550SNP
Higher age, female gender, osteoarthritis and blood transfusion protect against periprosthetic joint infection in total hip or knee arthroplasties: a systematic review and meta-analysis	Hip, knee	Y	Male gender, coagulopathy, alcohol abuse, surgical site infection (highest score), and high NNIS system surgical patient index score
Inadequate glycemic control is associated with increased surgical site infection in total joint arthroplasty: a systematic review and meta-analysis	Hip, knee, shoulder	Y	Inadequate glycemic control
Incidence and risk factors for surgical site infection following total knee arthroplasty: a systematic review and meta-analysis	Knee	N	Steroid use, bilateral surgery, drain usage, bone graft, urinary tract infection, hypertension, and rheumatoid arthritis
Incidence and risk factors for surgical site infection following total knee arthroplasty: a systematic review and meta-analysis	Knee	Y	Male gender, age, obesity, smoking, American Society of Anesthesiologists scale (ASA) > 2, operative time, transfusion, diabetes mellitus, obesity
Intra-articular steroid injections and risk of infection following total hip replacement or total knee replacement: a meta-analysis of cohort studies	Hip, knee	Y	Intra-articular steroid injections
Is hemoglobin A1c and perioperative hyperglycemia predictive of periprosthetic joint infection following total joint arthroplasty?: A systematic review and meta-analysis	Hip, knee	Y	High HbA1c and perioperative hyperglycemia
Meta-analysis shows that obesity may be a significant risk factor for prosthetic joint infections	Hip	Y	Obesity
Patient-related risk factors for periprosthetic joint infection after total joint arthroplasty: a systematic review and meta-analysis	NA	N	Age, high alcohol intake
Patient-related risk factors for periprosthetic joint infection after total joint arthroplasty: a systematic review and meta-analysis	NA	Y	Histories of diabetes, rheumatoid arthritis, depression, steroid use, and previous joint surgery
Positive culture during reimplantation increases the risk of reinfection in two-stage exchange arthroplasty despite administrating prolonged antibiotics: a retrospective cohort study and meta-analysis	Hip, knee	Y	Positive culture at reimplantation
Preoperative malnutrition negatively correlates with postoperative wound complications and infection after total joint arthroplasty: a systematic review and meta-analysis	Hip, knee	Y	Preoperative malnutrition
Risk factors for deep infection after total knee arthroplasty: a meta-analysis	Knee	Y	BMI, diabetes mellitus, hypertension, steroid therapy, rheumatoid arthritis
Risk factors for deep infection after total knee arthroplasty: a meta-analysis	Knee	N	Gender, osteoarthritis, urinary tract infection, fixation method, American Society of Anesthesiologists, bilateral operation, age, transfusion, antibiotics, bone graft
Risk factors for periprosthetic joint infection after hip or knee arthroplasty in mainland of China: a meta-analysis	Hip, knee	Y	Diabetes mellitus, long-term use of steroids, long operation time (> 90 min), age (> 65 years), and previous history of hip or knee surgery
Risk factors for periprosthetic joint infection after total joint arthroplasty: a systematic review and meta-analysis	NA	Y	Body mass index, diabetes mellitus; corticosteroid therapy, hypoalbuminemia, history of rheumatoid arthritis, blood transfusion, presence of a wound drain, wound dehiscence, superficial surgical site infection, coagulopathy, malignancy, immunodepression, National Nosocomial Infections Surveillance Score ≥ 2, other nosocomial infection, prolonged operative time, previous surgery
Risk factors for periprosthetic joint infection after total joint arthroplasty: a systematic review and meta-analysis	NA	N	Cirrhosis, hypothyroidism, urinary tract infection, illicit drug abuse, alcohol abuse, hypercholesterolemia, hypertension, ischemic heart disease, peptic ulcer disease, hemiplegia or paraplegia, dementia, operation performed by a staff surgeon (vs. a trainee)
Risk of surgical site infection in patients with asymptomatic bacteriuria or abnormal urinalysis before joint arthroplasty: systematic review and meta-analysis	Hip, knee	Y	Asymptomatic bacteriuria
Tobacco use and risk of wound complications and periprosthetic joint infection: a systematic review and meta-analysis of primary total joint arthroplasty procedures	Hip, knee	Y	Tobacco
Total joint arthroplasty following intra-articular steroid injection: a literature review	Hip, knee	N	Intra-articular steroid injections

***NA*** not available, ***N*** the present study supported the topic not to be a risk factor of PJI, ***Y*** the present study supported the topic not to be a risk factor of PJI

**Table 12:** Prevention of PJI based on meta-analysis research

Title	Location	Y/N	Topic
Negative pressure wound therapy in total hip and knee arthroplasty: a meta-analysis	Hip, knee	Y	Negative pressure wound therapy
A systematic review and meta-analysis of antibiotic-impregnated bone cement use in primary total hip or knee arthroplasty	Hip, knee	Y	Antibiotic-impregnated bone cement
Antibiotic bone cement’s effect on infection rates in primary and revision total knee arthroplasties	Knee	N	Antibiotic-impregnated bone cement
Antibiotic prophylaxis for wound infections in total joint arthroplasty: a systematic review	Hip, knee, and other undefined location	Y	Antibiotic prophylaxis
Antibiotic-impregnated bone cement for preventing infection in patients receiving primary total hip and knee arthroplasty: a meta-analysis	Hip, knee	Y	Antibiotic-impregnated bone cement
Control strategies to prevent total hip replacement-related infections: a systematic review and mixed treatment comparison	Hip	Y	Systemic antibiotic prophylaxis in conjunction with antibiotic-impregnated cement and conventional ventilation
Efficacy of antibiotic-impregnated cement in total hip replacement: a meta-analysis	Hip	Y	Antibiotic-impregnated bone cement
Efficacy of prophylactic cefazoline and vancomycin in hip and knee surgery: a systematic review and meta-analysis	Hip, knee	Y	Antibiotic prophylaxis
Lack of efficacy of prophylactic application of antibiotic-loaded bone cement for prevention of infection in primary total knee arthroplasty: results of a meta-analysis	Knee	N	Antibiotic-impregnated bone cement
Perioperative antibiotic prophylaxis in total joint arthroplasty: a systematic review and meta-analysis	Hip, knee	N	Postoperative antibiotic prophylaxis or continuation beyond 24 h
Postoperative antibiotic prophylaxis in total hip and knee arthroplasty: a systematic review and meta-analysis of randomized controlled trials	Hip, knee	N	Postoperative antibiotic prophylaxis
Preoperative bathing with chlorhexidine reduces the incidence of surgical site infections after total knee arthroplasty	Knee	Y	Chlorhexidine
Preoperative chlorhexidine reduces the incidence of surgical site infections in total knee and hip arthroplasty: a systematic review and meta-analysis	Hip, knee	Y	Chlorhexidine
Prophylaxis with nasal decolonization in patients submitted to total knee and hip arthroplasty: systematic review and meta-analysis	Hip, knee	Y	Prophylaxis with nasal decolonization
Systematic review and meta-analysis of randomized controlled trials of antibiotics and antiseptics for preventing infection in people receiving primary total hip and knee prostheses	Hip, knee	N	Antibiotics and/or antiseptics
The hidden cost of commercial antibiotic-loaded bone cement: a systematic review of clinical results and cost implications following total knee arthroplasty	Knee	N	Antibiotic-impregnated bone cement

*N* the present study did not support the topic to be an effective prevention measure for PJI, *Y* the present study supported the topic to be an effective prevention measure for PJI

##### Comparative analysis

There were 26 comparative analytic studies from all meta-analyses, with most related to the hip and knee (11), followed by the hip as well as the hip and knee (7 each). There was no statistical difference found in 13 comparison studies (Table 13).

**Table 13:** Comparison studies of PJI based on meta-analysis

Title	Location	Topic			
Infection and revision rates following primary total knee arthroplasty in patients with rheumatoid arthritis versus osteoarthritis: a meta-analysis	Knee	Rheumatoid arthritis		Osteoarthritis	
Simultaneous versus staged bilateral total knee arthroplasty a meta-analysis evaluating mortality, peri-operative complications and infection rates	Knee	Simultaneous bilateral total knee arthroplasty	◯	Staged bilateral total knee arthroplasty	◯
Comparison of infection eradication rate of using articulating spacers containing bio-inert materials versus all-cement articulating spacers in revision of infected TKA: a systematic review and meta-analysis	Knee	Articulating spacers containing bio-inert materials		All-cement articulating spacers	
Comparison of the efficacy of static versus articular spacers in two-stage revision surgery for the treatment of infection following total knee arthroplasty: a meta-analysis	Knee	Articulating spacers	◯	Static spacers	◯
Do culture-negative periprosthetic joint infections have a worse outcome than culture-positive periprosthetic joint infections? A systematic review and meta-analysis	Hip, knee	Culture-positive infections	◯	Culture-negative infections	◯
Does cemented or cementless single-stage exchange arthroplasty of chronic periprosthetic hip infections provide similar infection rates to a two-stage? A systematic review	Hip	Single-stage exchange	◯	Two-stage exchange	◯
		Single-stage cementless	◯	Single-stage cemented	◯
Does simultaneous bilateral total joint arthroplasty increase deep infection risk compared to staged surgeries? A meta-analysis	Hip, knee	Staged bilateral total joint arthroplasty		Simultaneous bilateral total joint arthroplasty	
Dynamic versus static cement spacer in periprosthetic knee infection: a meta-analysis [Dynamischer vs. statischer Zementspacer in der Knietotalendoprotheseninfektion: Eine Metaanalyse]	Knee	Dynamic knee spacer	◯	Static knee spacer	◯
External fixation vs intramedullary nailing for knee arthrodesis after failed infected total knee arthroplasty: a systematic review and meta-analysis	Knee	External fixation	◯	Intramedullary nailing	◯
Implant fixation and risk of prosthetic joint infection following primary total hip replacement: meta-analysis of observational cohort and randomised intervention studies	Hip	Cemented fixations (plain and antibiotic combined, plain cemented fixations, hybrid fixations, reverse hybrid fixations)		Uncemented fixations	
Influence of fixation methods on prosthetic joint infection following primary total knee replacement: meta-analysis of observational cohort and randomised intervention studies	Knee	Cemented fixations (plain and antibiotic combined, plain cemented fixations, hybrid fixations, reverse hybrid fixations)		Uncemented fixations	
One- and two-stage surgical revision of infected elbow prostheses following total joint replacement: a systematic review	Elbow	Single-stage exchange	◯	Two-stage exchange	◯
One- and two-stage surgical revision of infected shoulder prostheses following arthroplasty surgery: a systematic review and meta-analysis	Shoulder	Single-stage exchange	◯	Two-stage exchange	◯
One- and two-stage surgical revision of peri-prosthetic joint infection of the hip: a pooled individual participant data analysis of 44 cohort studies	Hip	Single-stage exchange	◯	Two-stage exchange	◯
Postoperative deep infection after cemented versus cementless total hip arthroplasty: a meta-analysis	Hip	Cemented total hip arthroplasty		Cementless total hip arthroplasty	
Re-infection outcomes following one- and two-stage surgical revision of infected hip prosthesis: a systematic review and meta-analysis		Single-stage exchange (unselected patients)	◯	Two-stage exchange (unselected patients)	◯
Re-infection outcomes following one- and two-stage surgical revision of infected knee prosthesis: a systematic review and meta-analysis	Knee	Single-stage exchange (unselected patients)	◯	Two-stage exchange (unselected patients)	◯
Re-infection rates and clinical outcomes following arthrodesis with intramedullary nail and external fixator for infected knee prosthesis: a systematic review and meta-analysis	Knee	Arthrodesis with intramedullary nail		Arthrodesis with external fixator	
Use of static or articulating spacers for infection following total knee arthroplasty	Knee	Articulating spacers	◯	Static spacers	◯
The effect of wound dressings on infection following total joint arthroplasty	Hip, knee	Standard, absorbent dressings		Hydrofiber dressings	
Human immunodeficiency virus and total joint arthroplasty: the risk for infection is reduced	Hip, knee	HIV and hemophilia		HIV	
The impact of neuraxial versus general anesthesia on the incidence of postoperative surgical site infections following knee or hip arthroplasty a meta-analysis	Hip, knee	General anesthesia		Neuraxial anesthesia	
Tobacco use and risk of wound complications and periprosthetic joint infection: a systematic review and meta-analysis of primary total joint arthroplasty procedures	Hip, knee	Current tobacco users		Former tobacco users	

◯**:** The meta-analysis results showed that there was no significant relationship between the two topics. **:** The meta-analysis results demonstrated that the topic had a higher infection/reinfection rate, further compounded results, represented a risk factor, or was not an effective method of preventing infection; **:** The meta-analysis results showed that the topic had a lower infection rate/reinfection rate, more optimal result, represented an effective prevention measure against infection, or was not a risk factor

## Discussion

This bibliometric study presents 117 meta-analysis results from three databases (PubMed, Scopus, and Web of Science), with the greatest number of relevant papers in Scopus. Furthermore, we compared all databases with or without a search strategy, with PubMed demonstrating the greatest difference among the three databases. When combined with other databases, the missing information from the search strategy could be supplemented. All results could not be found with any of the databases, with or without a search strategy, whereas the combination of PubMed and Scopus enclosed all results without a search strategy. In addition, all available information from the database and search algorithm were collected and combined. Three to five database groups were found to comprise most options for meta-analysis. Embase, MEDLINE, and Cochrane were the top three most commonly used databases and were also mostly used for meta-analysis. The available search algorithm exported from 102 publications provided a reference for scholars for a further literature search and study design.

Meta-analysis could offer a useful effective reference to support or refute controversial conclusions from multiple studies. The bibliometric analysis showed that the first meta-analysis appeared in 2007, with an increasing trend in the ensuing years. The growth number likely reflects the development of the subject with an academic dispute, and the International Consensus Meeting on PJI also indicated the presence of disparate opinions on the management of PJI [54]. The current study also presented China as having the greatest number of publications in meta-analyses. This may be attributed to the fact that Chinese physicians are placed under immense pressure to publish under the health-system reforms [55]. Furthermore, the Chinese Association of Orthopaedic Surgeons (CAOS) play close attention to infection after joint arthroplasty. CAOS, which comprises the Chinese prosthetic joint infection society, was established in 2018 and perform PJI research by multiple centers. In China, Beijing and Shanghai had the greatest number of publication of PJI meta-analysis than other cities and is most likely related to a larger number of research institution concentrated in both regions. Institutions from the UK had the largest number of publications, with the majority from the University of Bristol. Analysis of author information showed that at least two authors were required for meta-analysis, with the most frequent number of collaborators was four. In meta-analysis studies, Setor K. Kunutsor from the University of Bristol had the most publications as the first author.

In all meta-analysis papers, the Journal of Arthroplasty had the most number of relevant papers. With more than 20 citations, PLoS ONE had the greatest number of publications from the most cited publication list. The Journal of Clinical Medicine had the minimum time from receipt to acceptance. In addition, the bibliometric method report showed most articles to be received and accepted on Wednesday.

In the top 10 most popular keywords on PJI meta-analysis, two keywords were related to treatment and diagnosis, with two-stage exchange and alpha-defensin in the top 10. Three keywords were associated with the location of PJI, with the majority on the hip and knee. Identical results were also found in regard to the location, with the top three keywords knee, hip, and shoulder. The most frequently used software in the meta-analysis were STATA, REVMAN, and Meta-Disc.

Among the diagnosis list in meta-analysis studies, the synovial fluid test was the most frequently used preoperative examination (64%). The most popular diagnostic test applied in recent years was synovial fluid alpha-defensin and has been incorporated in the 2018 Musculoskeletal Infection Society (MSIS) definition as one of the minor criteria [56]. When compared with conventional diagnostic methods, such as ESR, CRP, synovial fluid culture, and synovial fluid PMN%, alpha-defensin showed better sensitivity, especially in cases receiving antibiotics before joint puncture [57, 58]. In recent years, synovial fluid alpha-defensin could be detected using two different methods. One assay is the enzyme-linked immunosorbent assay (ELISA), which is performed in a laboratory with results obtained within 24 h. The second assay is the lateral flow device, which rapidly detects infection within 20 min without the need for a laboratory. Accordingly, pooled results supported the higher sensitivity of the synovial fluid alpha-defensin ELISA compared to the lateral flow test [16, 20, 23, 27]. The current meta-analysis demonstrated synovial fluid alpha-defensin to have the highest sensitivity in the diagnosis of PJI. As it represents a non-microbiological test, it could be used as a reliable reference for intraoperative microbiological diagnosis. Preoperative tests with the lowest sensitivities were synovial fluid gram staining (GS), synovial fluid procalcitonin (PCT), serum white blood cells (WBCs), and serum PCT, which were all found to have a sensitivity of less than 60%.

Sonicate fluid and periprosthetic tissue were performed most intraoperatively, whereas tissue PCR and sonicate fluid BCB were the most sensitive tests in tissue and sonicate fluid, respectively. In 2013, Qu et al. [37] performed the first meta-analysis of PCR in the diagnosis of PJI. The authors found that the tissue PCR had a higher sensitivity than synovial fluid PCR and sonicate fluid PCR (95% vs. 84% vs. 81%, respectively). However, tissue PCR showed the lowest specificity compared to synovial and sonicate fluid PCR (81% vs. 89% vs. 96%, respectively). However, this is in contrast to the study by Huang and colleagues [59], in which tissue PCR had lower sensitivity of 34% and the highest specificity of 100% among the three types. Due to limited data and that the included studies on tissue PCR were performed between 1999 and 2012 [37], the diagnostic value of tissue PCR remains unclear. The meta-analysis of sonicate fluid BCB presented a sensitivity of 85% and a specificity of 86% [46]. Compared to the conventional culture of sonicate fluid, BCB culture was more sensitive in patients with or without antibiotics and also detected infection within a shorter time than normal medium sonicate fluid culture [60–63]. Yet, the drawback of sonicate fluid BCB was the rate of false-positives, which was caused by contamination during the inoculation procedure of BCB with sonicate fluid. Therefore, careful handling is required to minimize contamination [64, 65]. Tissue and tissue swab GS were the two least frequently applied intraoperative tests, with a sensitivity of less than 20%.

Diagnosis prior to reimplantation always posed difficulty. In the meta-analysis study by Lee and colleagues [52], tissue culture demonstrated the highest sensitivity before reimplantation, which was based on two included studies (82%). Another meta-analysis study by Bian and co-workers [53] estimated the various tests during the first stage and/or predicted failed reimplantation beyond the second stage, with tissue culture showing a sensitivity of 30%, which was based on the results of nine studies. Synovial fluid PMN% demonstrated the highest sensitivity of 70% in the study by Bian et al., while the specificity was low at 71%. Interestingly, the author found that the spacer sonication fluid culture was the most accurate method with an area under the receiver operating characteristic curve of 0.8089. There was no single test that achieved an ideal result, with combined multiple tests to evaluate infection still required [53].

There were 40 meta-analyses related to risk factor and prevention, with the majority of articles on preventive measures focusing on systemic or location antibiotics use. In regard to the risk factor, most concerns focused on intra-articular steroid injections, followed by age, diabetes mellitus, and rheumatoid arthritis.

The top three comparison studies focused on cemented vs. cementless total joint arthroplasty, the outcome of using different types of spacers, and the outcome of one-stage vs. two-stage exchange. Cemented fixations were revealed to increase the overall PJI risk in comparison to uncemented fixations [66–68]. Interestingly, there was no significant difference in the eradication rate between articulating and static spacers in the infected knee replacement [69, 70]. The current meta-analysis supports that the infection control or reinfection rate of one-stage or two-stage exchange did not significantly differ in the hip, knee, elbow, and shoulder [71–75].

There are several limitations to the present study. First, the database of present bibliometric analyses was collected from three databases. Compared with results from without the search strategy, several articles were missed when using the search strategy, especially in PubMed. However, working with multiple databases could reduce this problem. In addition, we also collected database information from all meta-analyses. Embase, MEDLINE, and Cochrane were the most widely used databases. However, whether these databases were appropriate for bibliometric analysis remains unclear and requires further investigation. Second, due to the export of all meta-analysis information between different databases with disparate formats, a visualized analysis could not be performed. Third, although meta-analysis results on diagnosis, risk factors, prevention, and comparative studies were shown, the heterogeneity and quality of included meta-analysis studies were not considered. In the subgroup diagnosis, since there is no gold standard for the diagnosis of PJI, different culture results are obtained from the various diagnostics tests. The pooled sensitivity and specificity of meta-analysis are then further affected by potential false positive or negative results. Fourth, the current study only presented meta-analysis results and did not reflect the complete perspective of PJI research. The overall trends in this field are required to further confirm.

## Conclusion

The bibliometric analysis that presented global PJI research of meta-analysis studies showed an increasing trend between 2007 and 2019. The Embase database and STATA software were most frequently used for meta-analysis. Most studies focused on the periprosthetic hip and knee. The diagnostic alpha-defensin test, preventive measures on antibiotics use, and risk factors associated with intra-articular steroid injections were the most popular topics in recent years.

## Supplementary information

**Additional file 1:.** Supplementary S1.

## Data Availability

Data was extracted from references.

## Abbreviations

AGS: Antigranulocyte scintigraphy
BCB: Blood culture bottles
CAOS: Chinese Association of Orthopaedic Surgeons
CI: Confidence interval
CRP: C-reactive protein
CT: Computed tomography
ELISA: Enzyme-linked immunosorbent assays
ESR: Erythrocyte sedimentation
GS: Gram staining
LE: Leukocyte esterase
IL: Interleukin
PCR: Polymerase chain reaction
PCT: Procalcitonin
PET: Positron emission tomography
PJI: Periprosthetic joint infection
PMN%: Polymorphonucleocytes percentage
Sen: Sensitivity
Spe: Specificity
MeSH: Medical subject headings
MSIS: Musculoskeletal Infection Society
WBCs: White blood cells
WCC: White cell count
