# Supplementary material for: Meta-analysis in periprosthetic joint infection: a global bibliometric analysis
Source: J Orthop Surg Res. 2020 Jul 10;15:251. doi: 10.1186/s13018-020-01757-9 (PMC7350679; doi:10.1186/s13018-020-01757-9)
Supplement: Supplementary file 1 — Additional file 1:. Supplementary S1. [file 13018_2020_1757_MOESM1_ESM.pdf]

PJI-related keywords (196)

Adhesins;ankle;arthroplast*;arthroplastic;arthroplasties;arthroplasty;aseptic loosenin
g;aseptic loosening of joint prosthesis;bacteraem*;bacterem*;Bacteria;bacteria infe
-ction;bacteria infections;bacteria*;Bacterial Adhesion;bacterial infections;bacterie;
biofilm*;Biofilms;complication;deep infection;deep prosthetic infection;deep surgi-
cal site infection;ELBOW;endoprostheses;endoprosthesis;endoprosthetic;failed;faile
-d total joint arthroplasty;femoral;Femur;femur*;finger;graft;HA;hemiarthroplasty;
Hip;Hip arthroplastic;Hip arthroplasties;Hip arthroplasty;Hip implant;Hip implant-
s;Hip infection;hip joint;Hip joint replacement;Hip joint replacements;Hip operati
-on;Hip prostheses;Hip Prosthesis;hip replacement;Hip replacements;HSA;Implant
infection;implant*;Implantation;Implantations;Implants;infect;infect*;infected;infect
-ed hip prostheses;infected knee prostheses;infected total knee arthroplasty;infecti
-on;infection*;infections;infectious;infective;inflamed;inflammat*;inflammation;infla
-mmation*;joint;joint arthroplasty;joint infection;joint prostheses;joint prosthesis;J-
oint Prosthesis Implantation;Joint prosthesis implantations;joint prosthesis infectio
-n;joint replacement;joint replacements;knee;knee arthroplast*;Knee arthroplastic;k
-nee arthroplasty;knee infection;knee joint;knee prosthesis;Knee reconstruction;kn-
ee replacement;knee replacement*;Knee replacements;knee revision failure;knee r
-evision infection;knee surgery;metal-on-metal;Metal-on-Metal Joint Prostheses;op
-erative;"operative surgical procedures;"orthopaedic implant infection;orthopaedic-
s;orthopedic;orthopedics;peri implant infection;peri prosthetic joint infection;perip
-rosthetic; periprosthetic hip infection;periprosthetic infection;peri-prosthetic infect
-ion;periprosthetic infections;periprosthetic joint infection;Peri-prosthetic joint infe
-ction;periprosthetic joint infections;peri-prosthetic joint infections;periprosthetic k
-nee infection;PJI;PJIs;postoperative;postoperative post-operative;PPI;primary knee
infection;primary TKA infection;prosthe*;prostheses;Prostheses and Implants;pro
-sthesis;Prosthesis Implantation;Prosthesis Implantations;prosthesis infection;prosth
-esis infections;Prosthesis Related Infections;prosthesis-related;Prosthesis-related i-
nfection;prosthesis-related infection*;prosthesis- related infections;prosthetic;Prosthe
-tic hip infection;prosthetic infection;prosthetic joint infection;prosthetic joint inf-
ections;replac*;Replaced hip;replacement;Replacement Arthroplasties;Replacement
Arthroplasty;replacements;resection arthroplasty;reverse total shoulder arthroplast
-y;revision;revision arthroplasty;revision TKA infection;revision total hip arthropl
-asty;revision total hip replacement;RTSA;sepsi*;Sepsis;sepsis in revision total jo
-int arthroplasty;septi*;Septic;septic loosening;septically;Shoulder;shoulder arthropl
-asty;shoulder replacement;specific joint-associated procedures;SSI;surgery;surgical
infection;surgical procedures operative;surgical site infection;Surgical Wound D-
ehiscence;Surgical Wound Infection;THA;THR;Tibia;tibia*;TJA;TKA;TKR;total hi
-p;Total hip arthroplasty;total hip prosthesis;total hip replacement;total hip replac
-ement;total joint;Total Joint Arthroplasty;total joint replacement;Total joint repla
-cements;total knee arthroplasty;Total Knee Prosthesis;total knee replacement;tota
-l shoulder arthroplasty;traumatology;TSA;wound;Wound Infection

Diagnose (179)

111in;16S amplicon targeted sequencing;18F;18FDG;2deoxyglucose;2-fluoro-2-deo
xy-D-glucose;99m;99mtc;99mTc-HDP;99mTc-hydroxymethane diphosphonate;99m
Tc-MDP;99mTc-methylene diphosphonate;a-defensin;alfa-defensin;alpha defensin;a
-lpha Defensins;alpha-defensin;Alpha-Defensins;antigranulocyte scintigraphy;anti-g
ranulocyte scintigraphy;antimicrobial peptide;Antimicrobial Peptides;arthrograph*;
aspirate;aspiration;beta-Defensins;biomarker*;blood culture bottles;blood culture s-
ystem;blood culture vials;bone;BW 250/183;chemical shift;compute*;C- reactive;C
-Reactive Protein;CRP;CT;CTs;defensin;Defensins;Deoxyglucose;deoxy-glucose;dia
-gnose;diagnosing;diagnosis;diagnosis of prosthetic joint infection;diagnostic;diagn
-ostic test;echograph*;echotomograph*;electron beam;Emission-Computed;F-18DG;
Fab;FDG;fine needle aspiration;fluid;fluorin*;fluoro;Fluorodeoxyglucose;Fluorodeo
-xyglucose F18;fluoroscop*;fMRI*;frozen section analysis;frozen section biopsy;
Gamma Camera Imag*;genetic diagnosis;genomics;gram;histological evaluation;hi
-stology;histopathology;IL-6;imag*;image;image subtraction*;images;imaging;in-11
1;indium;inflammatory;inflammatory markers;interleukin;interleukin-6;intra- articul
ar;intra operative frozen section;intraoperative;joint aspiration;lateral flow test;L-
E;leucocyte;leukocyte esteras;leukocyte esterase;leukocyte scintigraphy;Leukoscan;
-magnetic resonance;Magnetic Resonance Imaging;Magnetic Resonance Spectrosc
-opy;Magnetisation*;Magnetization*;marker*;metagenomic sequencing;monoclonal
antibodies;monoclonal antibody;MR imag*;MRI*;Neutrophil;Neutrophil Antimicr
-obial;neutrophil antimicrobial peptide;Neutrophil Antimicrobial Peptides;next gen
-eration sequencing;NGS;NMR*;pathology of joint tissues;PCR;PCT;peptide neut
-rophil antimicrobial;Peptides;periprosthetic tissue;PET;PMN;polymerase chain rea
-ction;polymorphonuclear;positron emission tomography;positron-emission tomogr
aphy;presence of polymorphonuclear leukocytes;procalcitonin;protein;radiograph*;
Radiography;radioisotop*;radionuclid*;radionuclide imaging;Radiopharmaceuticals;r
-oentgen*;rontgen*;scan;scintigraph*;scintigraphy;scintiphotograph*;scintiscan*;sens
-itivity;septic;septically;serum;shotgun metagenomics;sonicate;sonicate fluid;sonicat
-ion;sonication fluid;sonograph*;specificity;stain;sulesomab;synovasure;synovial;syn
-ovial fluid;synovial fluid count;test;three-phase;tissue;Tomodensitometr*;tomograp
-h*;Tomography;triple-phase;ultraso*;ultrasonicate;ultrasonography;ultrasound;WBC
-scintigraphy;white cell;white cell count;x-radiograph*;X-Ray Computed;x ray*;x-
ray*;zeugmatograph*;Zonograph*

Outcome (60)

1-stage;2 stage;2-stage;antibiotic bone cement spacer;arthrodes;ARTHRODESIS;ar
-ticulating spacer;bone cement;bone cements;cemented;cementless;culture;Culture
negative;debridement;delayed reimplantation;direct exchange;direct-exchange;excha
-nge;Exchange arthroplasty;external;fixation;Fixator;Fusion;hybrid;Ilizarov;implant
retention;Nail;nail*;negative pressure;one- and two stage revision surgeries;one st
age;one-stage;one-stage revision;prosthesis exchange;reimplant$;reimplantation;RE
OPERATION;Replantation;reverse hybrid;revis$;revision;revision arthroplasty;rotat
-or cuff;simultaneous;single stage;single-stage;spacer;Spacers;stage reimplantation;
staged;staged reimplantation;staged revision;static Spacer;treatment outcome;two s
tage;two-stage;two-stage revision;uncemented;unilateral;wound

**Prevention (82)**
antibiotic cement;1 stage knee revision;2 stage knee revision;Air conditioning;Al -cohols; cement;anti infective;anti infective agents;antibacterial agent;antibacterial agents;Anti-Bacterial Agents;antibacterial*;antibiotic bone cement;antibiotic impr -egnated;antibiotic prophylactic;antibiotic prophylaxis;antibiotic*;antibiotic-impregn -ated bone cement;antibiotic-impregnated cement;Antibiotic-laden Cement;antibiot -ic loaded bone cement;antibiotic-loaded bone cement;Antibiotic-loaded Cement;a -ntiinfective agent;Anti-Infective Agents;antimicrobial;antimicrobial*;antimycobacte -rial agent;antimycobacterial agents;antiseptic*;Aztreonam;bone cement;bone ceme -nts;cefazolin;cefepime;cefuroxime;cement;cephalosporins;chloraprep;chlorhexidine; chlorhexidine-alcohol;chlorhexidineisopropyl;Ciprofloxacin;control;Controlled;conve -ntional operating room;conventional operating theatre;decolonization;Environmen -t;gentamicin;infection control;infection prevent*;infused bone cement;intravenous antibiotics;Iodophors;laden;laminar air flow;laminar air flow system;laminar airf -low;levaquin;linezolid;nasal decolonization;Ofloxacin;operating rooms;Operating t -heatre;Oxazolidinones;Povidone-Iodine;prevention;prevention and control;procedur -es;randomized controlled trial;surgical;surgical antimicrobial prophylaxis orthope -dic;systemic antibiotics;tobramycin;Trimethoprim-Sulfamethoxazole Combination; Turbulent air flow;ultra clean air;ultra-clean air;ultra-clean air system;Vancomyci -n;ventilation
**Risk factor (74)**
2 staged;2-stage;acquired immune deficiency syndrome;AIDS;albumin;anesthesia; Articulating surface;ASB;asymptomatic bacteriuria;asymptomatic leukocyturia;bact -eriuria;Bearing couples;Bearing surface;BMI;body mass index;Ceramic;China;cig-ar;cigarette;cigarettes;cigars;Cobaltchrome;comorbidity;culture;determinant;Diabetes mellitus;exchange;factor;gene;general anesthesia;genetic;Glycemic control;HbA1c; Hemoglobin A1C;hemophilia;HIV;human immunodeficiency virus;hyperglycemia; hypoalbuminemia;inject;Injection;intra-articular;malnutrition;Metal-on-metal;microbi -ology;neuraxial anesthesia;Nicotine;nutritional status;obesity;organism;Polyethylen -e;polymorphism;predictor;reimplant;reimplantation;risk;risk factor;smoker;smokers; Smoking;SNP;steroid;tobacco;Tobacco Products;transfusion;two staged;two-stage;U -HMWPE;urinalysis;urinary analysis;urinary tract infection;urine analysis;urologic diseases;UTI
